# The Role of microRNAs in Heart Failure: A Systematic Review

**DOI:** 10.3389/fcvm.2020.00161

**Published:** 2020-10-15

**Authors:** Ana Peterlin, Karolina Počivavšek, Danijel Petrovič, Borut Peterlin

**Affiliations:** ^1^Faculty of Medicine, Institute of Histology and Embryology, University of Ljubljana, Ljubljana, Slovenia; ^2^Department of Cardiovascular Surgery, University Medical Centre Ljubljana, Ljubljana, Slovenia; ^3^Clinical Institute of Genomic Medicine, University Medical Centre Ljubljana, Ljubljana, Slovenia

**Keywords:** heart failure, biomarker (BM), epigenetics (DNA methylation, histone modifications), microRNA (miR), systematic (literature) review

## Abstract

MicroRNAs are highly investigated for their role in the pathogenesis of cardiovascular diseases. Nevertheless, evidence for clinical implementation is still lacking. In our systematic review, we evaluated the potential of microRNAs as pathophysiological and diagnostic biomarkers of heart failure. We identified 72 differentially expressed microRNA molecules among groups of heart failure patients and control groups by searching the PubMed database. We did not identify a substantial overlap of differentially expressed microRNAs among different studies; only five microRNAs (miR-1228, miR-122, miR-423-5p, miR-142-3p, and exosomal miR-92b-5p) were differentially expressed in more than one included study. Gene set enrichment analysis, based on the gene targets of microRNAs presented in the included studies, showed that gene targets of differentially expressed microRNAs were enriched in the MAPK, TGFβ, PI3K-Akt, and IL-2 signaling pathways, as well as apoptosis pathway, p53 activity regulation, and angiogenesis pathway. Results of our systematic review show that there is currently insufficient support for the use of any of the presented microRNAs as pathophysiological or prognostic biomarkers in the clinical setting.

## Introduction

Heart failure is a major cause of morbidity and mortality worldwide. The prevalence of heart failure is on the rise, and it has been estimated that it will grow further and reach 10% of the general population in 2030 (1). It is defined as the inability of the heart to supply the peripheral tissues with a sufficient amount of blood and oxygen to meet their metabolic needs and is mainly a result of age-related cardiovascular conditions and associated changes in cardiovascular structure and function (1). Heart failure is predominantly caused by an underlying myocardial disease (especially myocardial ischemia); however, other cardiac diseases, including valve diseases, endocardial, or pericardial abnormalities and disorders in the heart rhythm, may also result in diminished cardiac function (2). Clinical and research criteria for heart failure are heterogeneous and prone to misclassification. Heart failure is usually diagnosed by physical examination, laboratory workup, cardiovascular imaging, and hemodynamic catheterization (3). One of the main challenges in diagnosing heart failure is the identification of reliable biomarkers. Natriuretic peptides are the most extensively studied and used biomarkers in heart failure (4). The serum brain natriuretic peptide is currently the only routinely used biomarker for heart failure with class 1A recommendation from both American and European guidelines (1, 5). Fibrosis markers galectin-3 and soluble suppression of tumorigenicity-2 have been included in the ACC/AHA guidelines (strength of recommendation 2b), but their clinical value is still uncertain (1, 5). In addition to proteins, circulating microRNAs gained significant interest as potential novel heart failure biomarkers.

MicroRNAs (miRNAs) are small (~22 nucleotides long) endogenous non-coding RNAs that play an important role by regulating post-transcriptional gene expression. They act to either inhibit the translation of messenger RNA or to induce the degradation of specific mRNA (6–8). MiRNAs circulating in the blood were found to be protected from degradation caused by endogenous RNases. Evidence suggests that resistance to degradation is achieved by binding of miRNA to carrier molecules, such as Argonaute 2, nucleophosmin, and lipoproteins (HDL), and by the packaging of miRNA in microparticles such as exosomes, microvesicles, or apoptotic bodies (9–11). Stability in blood, differential expression in healthy tissue vs. pathologically changed tissue, and robust laboratory methods that detect the expression of miRNAs with a high degree of sensitivity and specificity all make miRNAs good candidates as biomarkers of the disease (12).

Differentially expressed miRNA patterns were found to be associated with various pathophysiological mechanisms of heart failure, such as cardiac remodeling, hypertrophy, apoptosis, and hypoxia (13–32). The involvement of miRNAs in various pathophysiological mechanisms of heart failure, diverse subtypes of heart failure investigated in studies, different design and methods of the studies, and the difference in inclusion/exclusion criteria for patient selection, however, mean that their role in heart failure and their potential as biomarkers remains elusive (33).

In this review, we aim to provide an overview of current scientific support for the use of miRNAs as pathophysiological and diagnostic biomarkers for heart failure.

## Methods

### Search Strategy

The literature search was conducted in the PubMed database until August 2019, using the following terms: (“MiRNAs” OR “microRNAs”) AND (“heart failure” OR “HF”) in the title/abstract. We limited our search to articles written in English. The “AND” operator was used to create all possible combinations of selected terms. The literature search was conducted independently by two authors (AP and KP) who reached consensus on all of the research papers.

### Study Selection and Data Collection

Research papers were initially retrieved as title and abstract and screened for eligibility. All selected research papers were then retrieved as complete manuscripts and checked for compliance with inclusion and exclusion criteria. We included human studies meeting the following criteria: (1) heart failure diagnosis based on clinical features and confirmed with echocardiography; (2) the patients had measured specific miRNA expression level at the time of heart failure exacerbation/follow up examination; (3) case–control study design; (4) qPCR, real-time PCR, microarray, and RNAseq are acceptable methods to evaluate the expressions of miRNAs. The exclusion criteria applied to the studies were as follows: (1) patients had received medications before blood/serum samples were collected; (2) heart failure as an immediate consequence of acute myocardial infarction; (3) heart failure studied only on subpopulations of patients, i.e., diabetics; (4) research papers that were not focused on heart failure but were focused only on the specific pathophysiological mechanism leading to heart failure (i.e., hypertension, atherosclerosis, arrhythmias); and (5) the same cohort was already studied in other research papers. Newcastle-Ottawa quality assessment scale (34) was used to assess the quality of included research papers. For each research paper included in the systematic review, the following data were extracted: authors, year of publication, study population geographical origin, number of heart failure patients and controls, selected miRNAs, sample source, and employed diagnostic criteria for heart failure. Additionally, we checked for the information on performed normalization of the differential microRNA expression.

### Bioinformatic Analysis/Gene Set Enrichment Analysis

Using miRTarBase (release 8.0 beta), we identified all known gene targets for selected miRNAs that were differentially expressed in the research papers included in the systematic review (35). We then developed the list of all target genes related to 72 miRNAs identified in Table 1 and performed gene set enrichment analysis using Enrichr (36, 37). Results from KEGG, BioPlanet, and Panther databases were analyzed.

**Table 1:** Characteristics of research papers included in the systematic review.

**References**	**Country**	**Enrolled patients**	**Enrolled controls**	**Study design**	**Selected miRNAs**	**Sample source**	**Diagnostic criteria**
**Sample source: serum, plasma**							
Wang et al. (15)	China	10 HF patients	10 healthy age- and sex-matched controls	HyF	miR-26b-5p miR-8485 miR-940	Serum	2017 ACC/AHA/HFSA focused update guideline for the management of HF
Wu et al. (23)	China	28 HFrEF patients	30 healthy age- and sex-matched controls	TA	exo-miR-92b-5p	Serum	AHA and ESC guidelines
Guo et al. (16)	China	94 CHF patients: NYHA II (32), NYHA III (32), NYHA IV (30)	31 healthy age- and sex-matched controls	TA	miR-133a	Plasma	2009 Focused update: ACCF/AHA guidelines for the diagnosis and management of HF in adults
Li et al. (17)	China	96 AHF patients, NT-proBNP levels ≥1,000 ng/L	20 healthy controls and 96 patients with cardiovascular disease but without HF and NT-proBNP level below the age-related cutoff point	TA	miR-302b-3p	Serum	National heart failure diagnosis and treatment guidelines established by our national society of cardiology
Wu et al. (14)	China	43 DCM-AHF patients	34 age and sex-matched healthy controls	TA	exo-miR-92b-5p	Serum	AHA guidelines
Chen et al. (18)	China	46 HF patients, LVEF ≥50%: 13 patients for initial genome-wide microarray, 33 patients for the RT-qPCR validation step	23 healthy controls: 3 controls for initial genome-wide microarray, 20 controls for the RT-qPCR validation step	HyF	miR-3135b miR-3908 miR-5571-5p	Serum	Echocardiography
He et al. (19)	China	124 HF patients (8 IHF and 8 NIHF patients for initial RNA sequencing, 60 IHF and 48 NIHF for RT-qPCR validation)	43 healthy controls (8 controls for initial RNA sequencing, 35 controls for RT-qPCR validation)	HyF	miR-195-3p	Plasma	ACC/AHA guidelines
Scrutinio et al. (20)	Italy	64 patients: 10 patients for genome-wide serum miRNA expression analysis (5 moderate HF, 5 advanced HF) AND 54 patients for RT-qPCR validation (25 moderate HF patients, 29 advanced HF patients)	20 healthy controls: 5 controls for genome-wide serum miRNA expression analysis AND 15 controls for RT-qPCR validation	HyF	miR-26a-5p miR-150-5p	Serum	ESC guidelines
Li et al. (22)	China	14 heart transplant tissue for miRNA microarray analysis, 45 patients for RT-qPCR validation	10 heart transplant tissue for miRNA microarray analysis, 45 patients for RT-qPCR validation	HyF	miR-660-3p miR-665 miR-1285-3p miR-4491	Heart tissue, serum	ACC/AHA guidelines
Wei et al. (13)	China	32 HF patients: 18 NYHA III, 14 NYHA IV	32 individuals with healthy sinus rhythm	TA	miR-126	Plasma	NYHA classification, ECG
Cakmak et al. (24)	Turkey	42 systolic grade C HF patients: 20 NYHA II, 22 NYHA III or IV	15 age- and sex-matched healthy controls	HyF	miR-21 miR-650 miR-744 miR-516-5p miR-1292 miR-182 miR-1228 miR-595 miR-663b miR-1296 miR-1825 miR-299-3p miR-662 miR-122 miR-3148 miR-129-3p miR-3155 miR-3175 miR-583 miR-568 miR-30d miR-200a miR-1979 miR-371-3p miR-155 miR-502-5p	Serum	2009 Focused update: ACCF/AHA guidelines for the diagnosis and management of heart failure in adults.
Akat et al. (25)	USA	24 advanced HF patients, 14 stable HF patients	13 healthy controls	TA	miR-208b miR-499 miR-1-1 miR133-b	Plasma	Echocardiography, NYHA classification
Fan et al. (27)	China	45 HF patients with DCM	39 healthy age and sex-matched controls	TA	miR-423-5p	Plasma	NYHA classification and echocardiography and LVEF <45%
Fukushima et al. (30)	Japan	33 ischemic HF patients	17 healthy controls	TA	miR-126	Serum	NYHA classification
Tijsen et al. (32)	Netherlands	42 HF patients: 12 AHF patients for miRNA microarray analysis, 30 HF patients for RT-qPCR validation	39 healthy controls	HyF	miR-18b miR-423-5p miR-675	Plasma	The Framingham criteria and NT-proBNP >1,000 ng/L
**Sample source: whole blood, PBMC**							
Yu et al. (21)	China	50 DCM patients	41 healthy age- and sex-matched controls	TA	miR-185	Whole blood	1995 WHO/ISFC criteria
Vogel et al. (26)	Germany	53 HFrEF patients, validation cohort: 14 HFrEF patients (whole blood), 10 HFrEF patients (serum)	39 healthy controls, validation cohort: 8 healthy controls (whole blood), 10 healthy controls (serum)	HyF	miR-122 miR-1228 miR-1231 miR-200b miR-519e miR-520d-5p miR-558 miR-622	Whole blood, serum	ESC guidelines, echocardiography LVEF <50% and NYHA classification
Endo et al. (28)	Japan	8 patients NYHA II and 5 patients NYHA III/IV	6 healthy controls	TA	miR-210	PBMC	NYHA classification
Nair et al. (29)	USA	8 patients DC, 10 patients stable DCM, 13 patients AHF	8 healthy controls	HyF	miR-454 miR-500a miR-500b miR-142-3p miR-1246 miR-124-5p	PBMC	Echocardiography
Voellenkle et al. (31)	Italy	7 patients NIDCM, 8 patients ICM, validation cohort: 17 patients NIDCM, 15 patients ICM	9 controls, validation cohort: 19 controls	TA	miR-107 miR-139 miR-142-5p miR-142-3p miR-29b miR-125b-5p miR-497	PBMC	NYHA classification and LVEF ≤ 36%

*HFrEF, heart failure with reduced ejection fraction; HF, heart failure; TA, targeted analysis; HyF, hypothesis-free; ACC/AHA/HFSA, American College of Cardiology/American Heart Association/Heart Failure Society of America; DC, diastolic dysfunction; DCM-AHF, dilated cardiomyopathy–acute heart failure; CHF, chronic heart failure; AHF, acute heart failure; AF, atrial fibrillation; DCM, dilated cardiomyopathy; LVEF, left ventricular ejection fraction; NYHA, New York Heart Association classification of heart failure; PBMC, peripheral blood mononuclear cells; NIDCM, nonischemic dilated cardiomyopathy; ICM, ischemic cardiomyopathy*.

## Results

**Figure 1:**
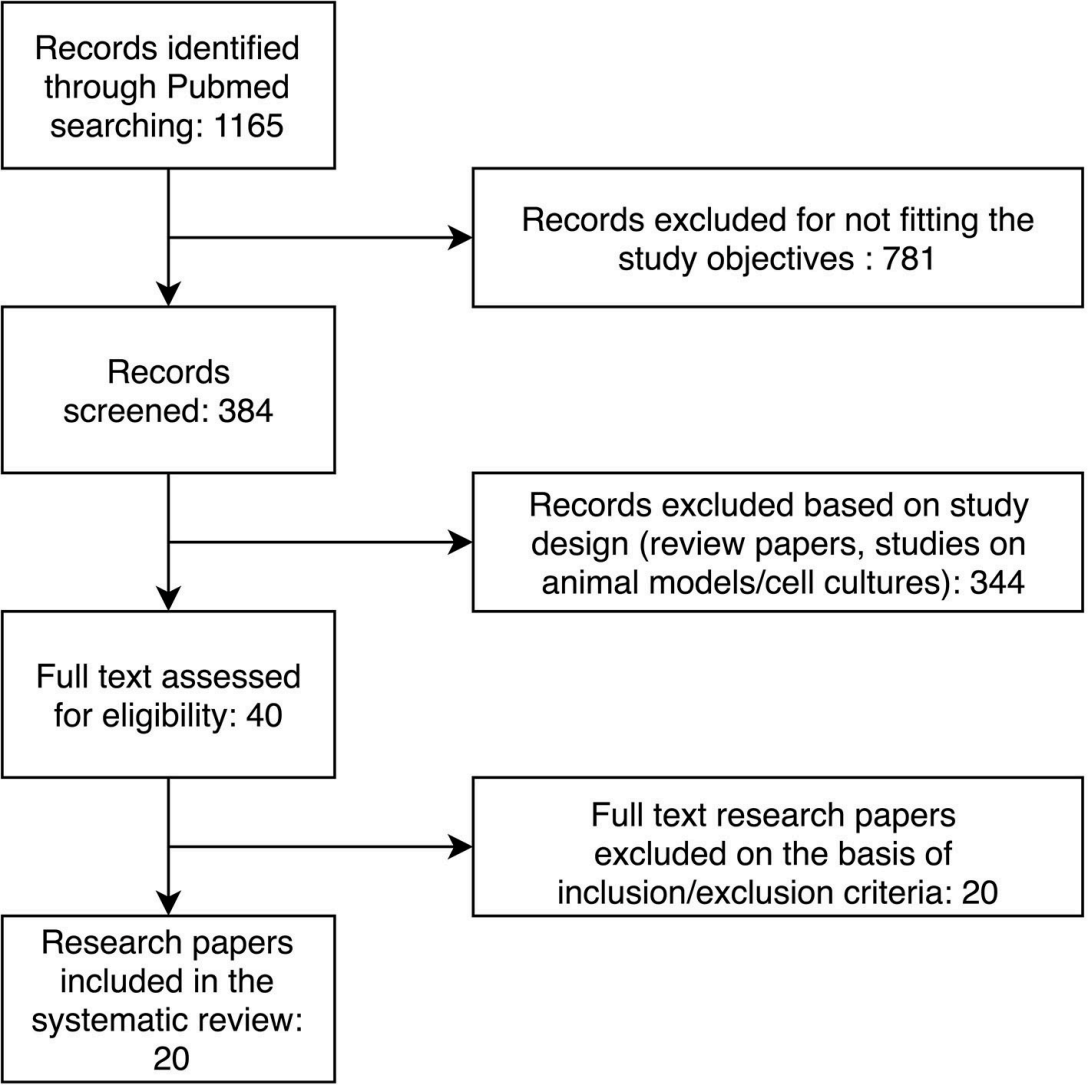
Flow diagram of the study selection process.

Through the initial keyword search, we retrieved 1,165 research papers. The further selection process is presented in Figure 1. After applying inclusion and exclusion criteria, we identified 20 research papers to include in our systematic review. The characteristics of the included studies are presented in Table 1. Selected studies described 72 differentially expressed miRNAs in patients with heart failure compared to controls without heart failure.

The Newcastle-Ottawa questionnaire was applied to assess the quality of the included studies (Table 2). The most common overall quality score was 7/9. Important shortcomings of studies included in the systematic review were suboptimal comparability of cases and controls as well as different definitions for cases and controls in the included studies.

**Table 2:** Quality assessment of included research papers using the Newcastle-Ottawa scale.

**Reference**	**Selection**				**Comparability**	**Exposure**			**Overall quality score**
	**An adequate definition of cases**	**Representativeness of cases**	**Selection of controls**	**Definition of controls**	**Comparability of cases and controls based on design or analysis**	**Ascertainment of exposure**	**Same method for ascertainment for cases and controls**	**Non-response rate**	
Wang et al. (15)	*	*	*	*	**	*	*	–	********
Wu et al. (2018)	*	*	*	*	**	*	*	–	********
Guo et al. (16)	*	*	*	*	**	*	*	–	********
Li et al. (17)	*	*	*	*	*	*	*	–	*******
Wu et al. (2018)	*	*	*	*	*	*	*	–	********
Chen et al. (18)	*	*	*	*	*	*	*	–	*******
He et al. (19)	*	*	*	*	*	*	*	–	*******
Scr]tinio et al. (20)	*	*	*	*	*	*	*	–	*******
Yu et al. (21)	*	*	*	*	**	*	*	–	********
Li et al. (22)	*	*	*	*	*	*	*	–	*******
Wei et al. (13)	*	*	*	*	*	*	*	–	*******
Cakmak et al. (24)	*	*	*	*	**	*	*	–	********
Akat et al. (25)	*	*	*	*	*	*	*	–	*******
Vogel et al. (26)	*	*	*	*	*	*	*	–	*******
Fan et al. (27)	*	*	*	*	**	*	*	–	********
Endo et al. (28)	*	*	*	*	*	*	*	–	*******
Nair et al. (29)	*	*	*	*	*	*	*	–	*******
Fukushima et al. (30)	*	*	*	*	*	*	*	–	*******
Voellenkle et al. (31)	*	*	–	–	*	*	*	–	*****
Tijsen et al. (32)	*	*	*	*	*	*	*	–	*******

*According to the Newcastle–Ottawa scale, a study can be awarded a maximum of one star for each numbered item within the Selection and Exposure categories. A maximum of two stars can be given for Comparability*.

The majority of included studies (17/18 RT-qPCR studies) provides information on performed normalization. However, there was a discrepancy in the normalization methods (exogenous or endogenous controls) and selected control microRNAs. The most commonly used control microRNAs were U6 snRNA, i.e., in seven studies (13–15, 19, 22, 23, 29, 32) and miR-39 in three studies (16, 20, 27).

By searching miRTarBase (35), we identified 2,052 potential gene targets for selected differentially expressed miRNAs and performed gene set enrichment analysis using Enrichr (36, 37). To reveal a pathophysiologically important set of genes, we investigated KEGG, BioPlanet, and Panther databases. The results are presented in Table 3.

**Table 3:** Top enriched pathways defined by gene targets of differentially expressed miRNA in studies investigating serum and plasma samples.

**KEGG 2019**	**BioPlanet 2019**	**Panther 2016**
Pathways in cancer	Pathways in cancer	p53 pathway feedback loops 2
Hepatitis B	Integrated breast cancer pathway	Apoptosis signaling pathway
AGE-RAGE signaling pathway in diabetic complications	Interleukin-2 signaling pathway	Angiogenesis
Colorectal cancer	Colorectal cancer	p53 pathway
MAPK signaling pathway	TGF- β signaling pathway	CCKR signaling map ST
TNF signaling pathway	ATM-dependent DNA damage response	Insulin/IGF pathway-protein kinase B signaling cascade
Measles	Prostate cancer	EGF receptor signaling pathway
Kaposi sarcoma-associated herpesvirus infection	p53 activity regulation	TGF-β signaling pathway
Prostate cancer	Androgen receptor signaling, proteolysis, and transcription regulation	Ras pathway
Human papillomavirus infection	Chronic myeloid leukemia	PI3 kinase pathway

## Discussion

We identified 72 differentially expressed miRNAs among groups of heart failure patients and control groups with a systematic review. Among 72 differentially expressed miRNAs, only 5 miRNAs, namely, miR-1228, miR-122, miR-423-5p, miR-142-3p, and exosomal miR-92b-5p were differentially expressed in more than one included study.

Two studies found increased levels of miR-1228 and miR-122 in patients with heart failure (24, 26). Vogel et al. found high expression of miR-1228 in CD15+ granulocytes and speculated that differences in leukocyte subpopulations might influence the inflammatory processes known to play an important role in the development and progression of heart failure (26, 26, 38–41). They found miR-122 to be among the most significantly up-regulated miRNAs with good discriminative power as a single marker for systolic heart failure (26). MiR-122 was previously investigated in the porcine cardiogenic shock model, where it was significantly up-regulated in blood samples (42).

Studies by Tijsen et al. and Fan et al. found miR423-5p to be significantly increased in heart failure patients compared to healthy controls and speculated that miR423-5p could be a significant predictor of heart failure diagnosis (27, 32). They also found that miR423-5p correlated with NT-proBNP (27, 32). While Tijsen et al. found a correlation between miR423-5p and NYHA classification, Fan et al. did not find plasma levels of miR423-5p to correlate with NYHA functional class or left ventricular ejection fraction values (27). Tijsen et al. did not specify the subpopulation of heart failure patients, while Fan et al. studied the subpopulation of heart failure patients with dilated cardiomyopathy. Goren et al. reported higher plasma levels of miR423-5p in patients suffering from heart failure due to dilated cardiomyopathy (43). MiR423-5p was up-regulated in array studies of failing human myocardium (44).

The results regarding the differential expression of miR142-3p were conflicting (29, 31). Vollenkle et al. found miR142-3p to be significantly increased in patients with non-ischemic dilated cardiomyopathy, while Nair et al. found miR142-3p to be downregulated in patients with heart failure due to dilated cardiomyopathy. Sample sources in both studies were peripheral blood mononuclear cells (29, 31).

Wu et al. investigated the role of serum exosomal miR-92b-5p in two different subpopulations of heart failure patients (patients with heart failure with reduced ejection fraction and patients with dilated cardiomyopathy caused by acute heart failure). They found increased expression levels of exo-miR-92b in both patient subgroups compared to control groups. Both studies also found miR-92b to be positively related to the left atrium diameter, left ventricular end-diastolic dimension, and left ventricular end-systolic dimension and negatively related to left ventricular ejection fraction and left ventricular fractional shortening (14, 23). Due to sequence similarity with miR-92a, which was studied in the mouse model, they predicted]that miR-92b could be involved in angiogenesis and functional recovery of ischemic tissues (45); however, they acknowledged the need of further basic research to prove the pathophysiological role of miR-92b in heart failure.

Through a thorough assessment of previously published studies, we established that the evidence of the magnitude of effect and certainty of the evidence, contextual factors including improvement of the predictive diagnostic value of investigated miRNAs, and pathophysiological action of miRNA in heart failure are still poorly investigated at present. Similarly, analytical and clinical validity, as well as clinical utility, have not been assessed yet (46, 47). Therefore, further studies are needed, which would follow the scientific statement on the criteria for the evaluation of novel markers of cardiovascular risk (48), which emphasize the importance of research design, representative at-risk population, adequate outcome events, and inclusion of measures of both discrimination and accuracy among others.

Based on the results of this systematic review, it is evident that none of the miRNAs could be considered to be used as a biomarker in the clinical setting. Therefore, we performed gene set enrichment analysis on 2,558 genes that were defined as targets of 72 differentially expressed miRNAs to investigate if there are any common enriched pathways related to pathophysiological processes of heart failure. We found support for an association of pathway enrichment results with heart failure for MAPK signaling pathway, TGFβ signaling pathway, PI3K-Akt signaling pathway, IL-2 signaling pathway, apoptosis pathway, p53 activity regulation, and angiogenesis pathway.

Cardiac angiogenesis, especially of microvasculature, in the setting of pathological cardiac hypertrophy, is insufficient to maintain adequate perfusion. Impaired production of NO from dysfunctional endothelial cells is one of the most researched pathophysiological mechanisms that impair angiogenesis (49). Inhibition of NO production in cardiomyocytes was shown to rapidly increase the production of reactive oxygen species, to activate p38 MAP kinase and enhance TGFβ and TNFα expression (50).

Mitogen-activated protein kinase (MAPK) signaling cascades are considered to be important regulators of cardiac hypertrophic response (51–53). Numerous reports have demonstrated that p38 MAPK inhibition can reduce cardiomyocyte growth in response to hypertrophic stimuli *in vitro* (54–57). Furthermore, chronic activation of the p38 MAPK pathway has been associated with the induction of hypertrophic response in cultured cardiomyocytes (55–57). Myocardial ischemia was also found to induce p38 MAPK activation, while p38 MAPK inhibition has been demonstrated to attenuate apoptosis in ischemia/reperfusion-injured hearts (58).

Several studies have already linked different miRNAs with the PI3K-Akt pathway (59–61). Two miRNAs, identified in our systematic review, were implied to have a role in the regulation of the PI3K-Akt pathway, namely, miR-200a-3p and miR302s. MiR-200a-3p accelerated cardiac hypertrophy by directly modulating WDR1 and simultaneously regulating PTEN/PI3K/AKT/CREB/WDR1 pathway (60), while the miR302-367 cluster was found to impair autophagy to worsen cardiac hypertrophy through silencing PTEN and consequently activating PI3K/AKT/mTOR pathway (59). Selective activation of distinct PI3K signaling pathways was also shown in a longitudinal canine model of heart failure and cardiac regeneration (62).

The results of previously published studies on the effects of IL-2 on heart-related pathologies are inconsistent. On the one hand, plasma levels of IL-2 were shown to be elevated in patients with acute myocardial infarction, angina pectoris, and dilated cardiomyopathy, while on the other hand, there are reports that suggest a potential therapeutic effect of IL-2 in the setting of acute myocardial infarction (63, 64).

TGFβ was found to be involved in cardiac remodeling (65). Specifically, numerous studies indicated that increased TGFβ1 expression plays an important role in heart hypertrophy, cardiac fibrosis, and cardiomyocyte apoptosis (66–70). Apoptosis importantly contributes to cardiomyocyte death in acute myocardial infarction (71, 72). A high grade of apoptosis is also present in the setting of unstable angina pectoris (73) and correlates with parameters of progressive left ventricular remodeling (74, 75). Increased p53 expression levels and other components of apoptosis pathways were reported in the myocardial tissue of patients with heart pathologies and were found to progressively increase in the process of heart failure (76–78). For example, it was shown that elevated levels of p53 in the myocardium, as a consequence of hypoxic stress due to acute myocardial infarction, led to the apoptosis of cardiomyocytes (79). P53 was also shown to be up-regulated due to the cardiotoxic effects on myocytes caused by doxorubicin treatment (80).

We discovered that many of the most enriched pathways were associated with different types of cancer, especially prostate cancer, colorectal cancer, and chronic myeloid leukemia. This discovery is not all that surprising since more than 50% of the miRNA genes are located in regions associated with cancer (81).

The systematic evaluation of diagnostic and prognostic values of miRNA presented in this study has some limitations due to different subpopulations of patients with various heart failure phenotypes across different studies, a small number of patients per study, and different technical approaches for miRNA identification and analysis (targeted sequencing or hypothesis-free approach and different methods of normalization). Furthermore, inferrals about a pathophysiological mechanism based on pathway analysis of circulating microRNA should be made with caution since heart tissue was not analyzed directly. Reporting bias may exist to some extent because we only included research papers written in English or because only positive results were published.

## Conclusions

The results of our systematic review on the role of miRNAs as pathophysiological and diagnostic biomarkers of heart failure show that there is currently insufficient support for the use of any of the presented miRNAs in the clinical setting. Gene set enrichment analysis showed that gene targets of differentially expressed miRNAs were enriched in pathways playing an important role in the MAPK, TGFβ, PI3K-Akt, PDGF, and IL-2 signaling pathways, as well as, apoptosis pathway, p53 activity regulation, and angiogenesis pathway. To establish the definite value of miRNAs as pathophysiological and diagnostic markers, future experimental studies employing the same methodological design and performed on large sample sizes are needed.

## Data Availability Statement

The datasets generated for this study are available on request to the corresponding author.

## Author Contributions

All authors listed have made a substantial, direct and intellectual contribution to the work, and approved it for publication.

## Conflict of Interest

The authors declare that the research was conducted in the absence of any commercial or financial relationships that could be construed as a potential conflict of interest.

## Abbreviations

miRNA/miR: microRNA
HF: heart failure
CHF: chronic heart failure
AHF: acute heart failure
HFrEF: heart failure with reduced ejection fraction
ACC/AHA/HFSA: American College of Cardiology/American Heart Association/Heart Failure Society of America
DC: diastolic dysfunction
DCM-AHF: dilated cardiomyopathy–acute heart failure
NIDCM: non-ischemic dilated cardiomyopathy
ICM: ischemic cardiomyopathy
DCM: dilated cardiomyopathy
LVEF: left ventricular ejection fraction
NYHA: New York Heart Association classification of heart failure
HDL: high-density lipoprotein
PCR: polymerase chain reaction
snRNA: small nuclear RNA
KEGG: Kyoto Encyclopedia of Genes and Genomes
NT-proBNP: N-terminal pro b-type natriuretic peptide
EGAPP Work Group: The Evaluation of Genomic Applications in Practice and Prevention Work Group
MAPK: mitogen-activated protein kinase
TGFβ: transforming growth factor beta
NO: nitric oxide
IL-2: interleukin 2
TA: targeted analysis
HyF: hypothesis-free
PBMC: peripheral blood mononuclear cells.
